# 2PE-STED Microscopy with a Single Ti: Sapphire Laser for Reduced Illumination

**DOI:** 10.1371/journal.pone.0088464

**Published:** 2014-02-06

**Authors:** Qifeng Li, Yang Wang, Da Chen, Sherry S. H. Wu

**Affiliations:** 1 College of Precision Instrument and Optoelectronics Engineering, Tianjin University, Tianjin, China; 2 Tianjin Key Laboratory of Biomedical Detecting Techniques and Instruments, Tianjin, China; 3 Department of Chemistry, University of British Columbia, Vancouver, British Columbia, Canada; Tufts University, United States of America

## Abstract

We reported a new effective approach to carry out two-photon excitation stimulated emission depletion (2PE-STED) microscopy using a single Ti:sapphire laser system. With an acoustic-optic Bragg cell, the modulated-CW 2PE STED microscope had the benefits of both CW and pulse approaches: lower input power, simple optical scheme and no complicated synchronization. Additionally, it also took advantages of fluorescence yield increasing. The sub-diffraction-limit resolution was demonstrated using ATTO 425-tagged clathrin-coated vesicles.

## Introduction

Fluorescence microscopy is one of the most powerful techniques available for biological studies. [1] However, because of the diffraction limit, the resolution of a far-field fluorescent microscope is typically no better than 200 nm. To visualize cellular structures smaller than 200 nm, scientists have been relying on electron microscopes (EM). Although EM has very high resolution, [2] it has many practical issues that limit its utility for biological studies. The interaction between the electrons and the sample prohibits the electrons from penetrating deep into the sample. [3] Therefore, a sample for EM must be fixed and thin-sectioned, which may result in artifacts. Recent developments in super resolution optical microscopy, such as stimulated emission depletion (STED) [4], photoactivated localization microscopy (PALM) [5], [6], stochastic optical reconstruction microscopy (STORM) [7], and structure illumination microscopy (SIM) [8] have achieved sub-diffraction-limit resolution. [9] While SIM, PALM, and STORM require mathematical reconstructions to obtain a high-resolution image, STED does not. However, setting up a STED microscope is costly because it involves two lasers at different wavelengths. Earlier STED microscopes used two synchronized trains of pulses: one excitation pulse of typically less than 100 ps duration followed by a 200 ps pulse for depletion. [10]–[12] In these setups, the depletion pulses in the visible region were typically generated in an optical parametric oscillator (OPO), stretched to 200 ps, and then synchronized with the excitation pulse. More recently, it was shown that STED microscopy can be implemented with CW lasers, simplifying the instrumental requirement for STED microscopy. [13].

Ti:sapphire lasers are one of the most popular lasers in research labs. It provides a cost effective possibility for two-photon excitation (2PE) CW-STED. A typical Ti:sapphire laser consists of a CW pump laser at 532 nm and a Ti:sapphire oscillator to generate femtosecond pulses in the range of 700–1000 nm. Previously, 2PE STED microscope has been demonstrated using two Ti:sapphire lasers. [14] The setup required the synchronization of two Ti:sapphire laser pulses: one for excitation and the other for pumping an OPO to generate a 580 nm depletion beam. Although the pulsed 2PE STED has been proved a powerful superresolving tool deep in living cell or tissue, [15], [16] the high cost and complexity of such a synchronized laser system would be prohibitive for most research labs. The single wavelength pulsed 2PE STED simplified the optical scheme if not considering the restricted fluorophore. [17] A different approach was to use a separate CW laser for depletion, [18], [19] but additional cost is also involved. Much more CW laser power yields poorer contrast, unexpected photon and photobleaching, [20] which is a challenge for living cell or tissue.

In this article, we demonstrate a new way to carry out 2PE STED microscopy by using the readily available 532 nm as the depletion beam. With a proper selection of fluorescence dye, the 2PE STED was demonstrated to image clathrin-coated vesicles with sub-diffraction-limited resolution. Additionally, a modulation technique was introduced to reduce the depletion laser power by three orders of magnitude for reduced photobleaching.

## Materials and Methods

### 2PE STED Microscopy

**Figure 1:**
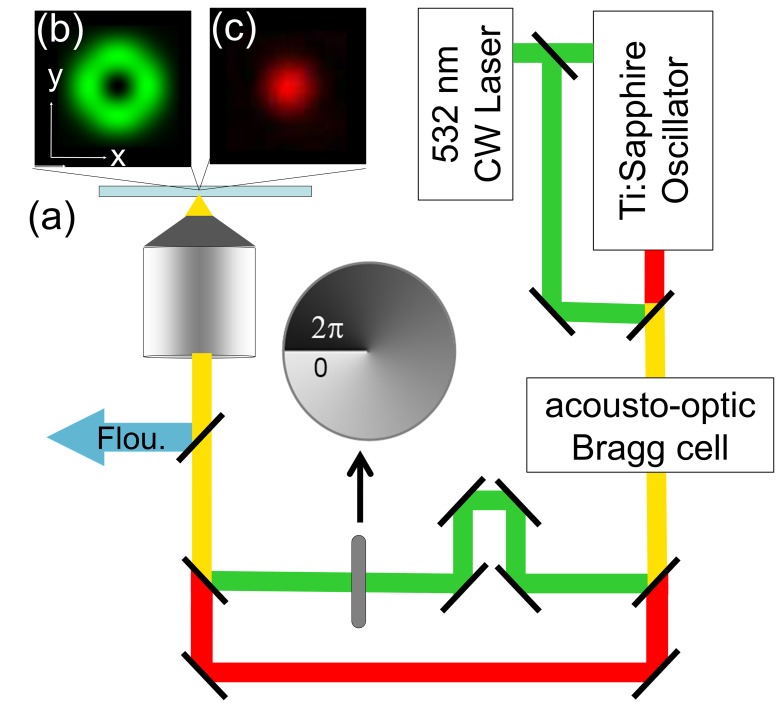
Experimental setup for 2PE-STED microscopy. (a) Schematic layout of modulated CW 2PE-STED microscopy. (b) The doughnut-shape intensity profile of the 532 nm depletion beam was obtained by using a spiral phase plate shown in (a). (c) The Gaussian intensity profile of the two-photon excitation beam at 860 nm.

The layout of the 2PE STED microscope is shown in Fig. 1a. The 2PE excitation was carried out using a 130-fs Ti:sapphire laser (MIRA 900, Coherent, USA) with a wavelength of 860 nm and a repetition rate of 76 MHz. The 532 nm depletion beam was obtained by splitting the 532 nm beam before the Ti:sapphire oscillator. The two beams were then combined using a dichroic mirror. The repetition rate was reduced to 0.25 MHz using an acousto-optic Bragg cell (coherent, USA). After the Bragg cell, the power were 0.036 mW for the 860 nm beam and 0.11 mW for the 532 nm beam. These two beams were then separated using a dichroic mirror to allow the 532 nm beam passing through a spiral phase plate (RPC photonics, USA) before these two beam were combined again. The spiral phase plate produced the doughnut-shaped focal intensity profile of the depletion beam shown in Fig. 1b. [21] The 2PE excitation spot (Fig. 1c) was then positioned to the center of the doughnut-shaped depletion beam. An 100X objective lens (NA 1.4 oil, HCX PL APO CS, Leica, Germany) was used for imaging. The fluorescence signal was recorded by an avalanche photodiodes (Micro Photon Devices, Italy) and a photon counter. All images were obtained with the samples mounted on a three dimensional piezo-scanning stage. While the laser beams were fixed, the piezo-scanning stage was scanned at 2 ms/pixel.

### Cell Culture and Immunocytochemistry

Chinese Hamster Ovary (CHO) cells were cultured in Alpha-MEM (Invitrogen Life Technologies, Carlsbad, CA) containing 10% FBS and 2 mM L-glutamine, at 37°C in a 5% CO2 incubator. The cells were fixed with 4% paraformaldehyde in phosphate buffered saline (PBS) for 10 min, and permeabilized with 0.1% Triton X-100 in PBS for 15 min. The cells were blocked with 1% BSA, 0.05% Tween 20 in PBS for 30 min, and incubated with rabbit anti-Clathrin heavy chain (Abcam, Cambridge, MA, 1∶500) antibody at 4°C overnight. ATTO 425 (Fluka, Buchs, Switzerland)-conjugated goat anti-rabbit IgG (Invitrogen Life Technologies, Carlsbad, CA) was used as the secondary antibody, and incubated with the cells for 1 hr. The cells were washed with PBS or 0.05% Tween 20 plus PBS for 5 min three times between each step. After washing with 0.1% BSA, 0.05% Tween 20 in PBS, The cells were mounted with mowiol (Sigma-Aldrich, Canada).

## Results

**Figure 2:**
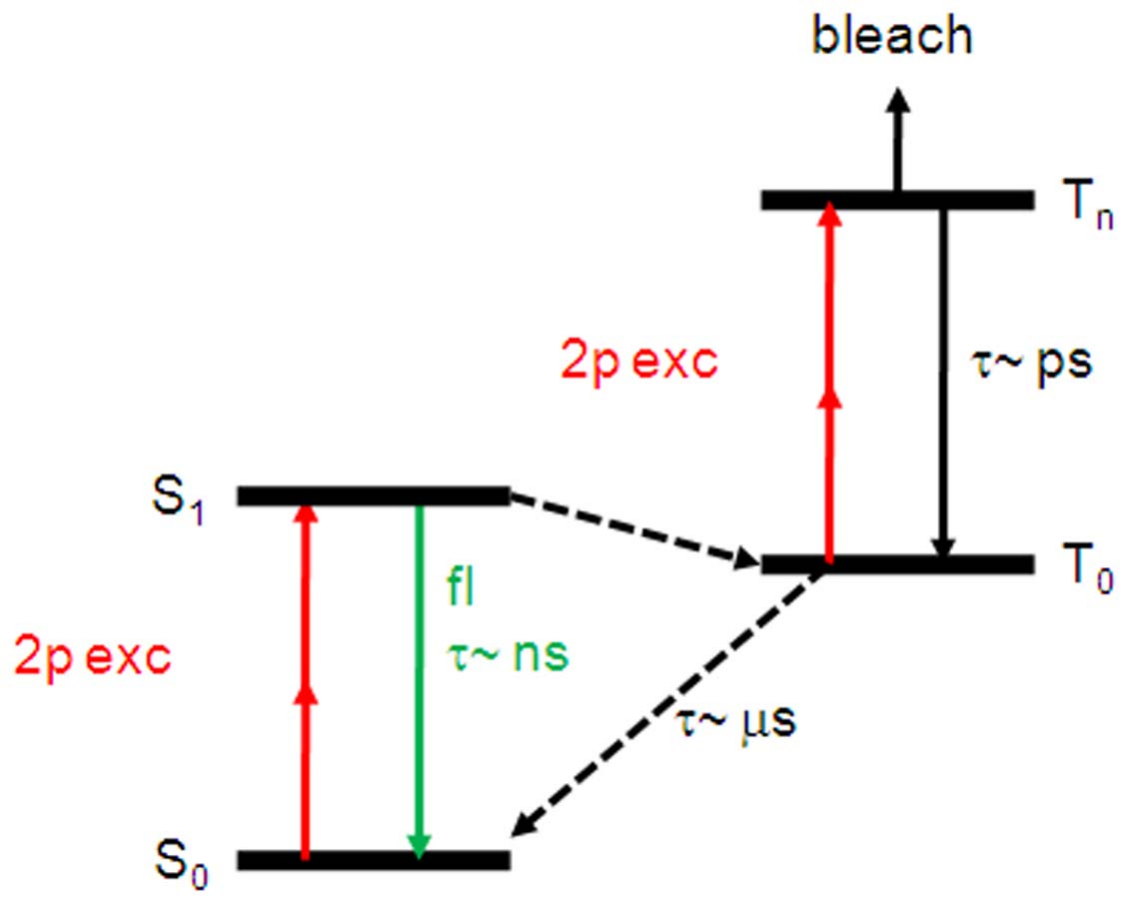
Energy diagram showing the photobleaching pathway via the triplet state. The fluorescence (fl) lifetime of the excited state S_0_ is typically a few to several ns. When trapped in the triplet state (T_0_), it will take ∼µs to relax to the ground state. Further excitations form the triplet states are more likely to bleach the fluorophore.

Previous studies have shown that the overall fluorescence yield significantly increase by reducing the repetition rate of a Ti:sapphire laser. [22] The repetition rate of a Ti:sapphire laser is typically around 80 MHz, which is equivalent to a pulse separation time Δt of 12.5 ns. As indicated in Fig. 2, 13 ns is longer than the fluorescence lifetime of most dyes, but it is about three orders of magnitude shorter than the lifetime of dark states, presumably the triplet states. It is not an ideal situation because illuminating molecules in the triplet states significantly increases the probability of photobleaching. It has been demonstrated that reducing the repetition rate below 1 MHz increased the total 2PE fluorescence yield by ∼25-fold for GFP and ∼20-fold for ATTO 532. [22] Although not necessary, the increased photon count is beneficial for obtaining STED images with a better signal-to-noise ratio. In the current study, the repetition rate of the Ti:sapphire laser was reduced, using an acousto-optic Bragg cell, from 76 MHz to 0.25 MHz, which increased the pulse separation time Δt from 13 ns to 4 µs and allowed the electrons in the triplet state to relax back to the ground state S_0_ before the next excitation pulse arrives. [22].

**Figure 3:**
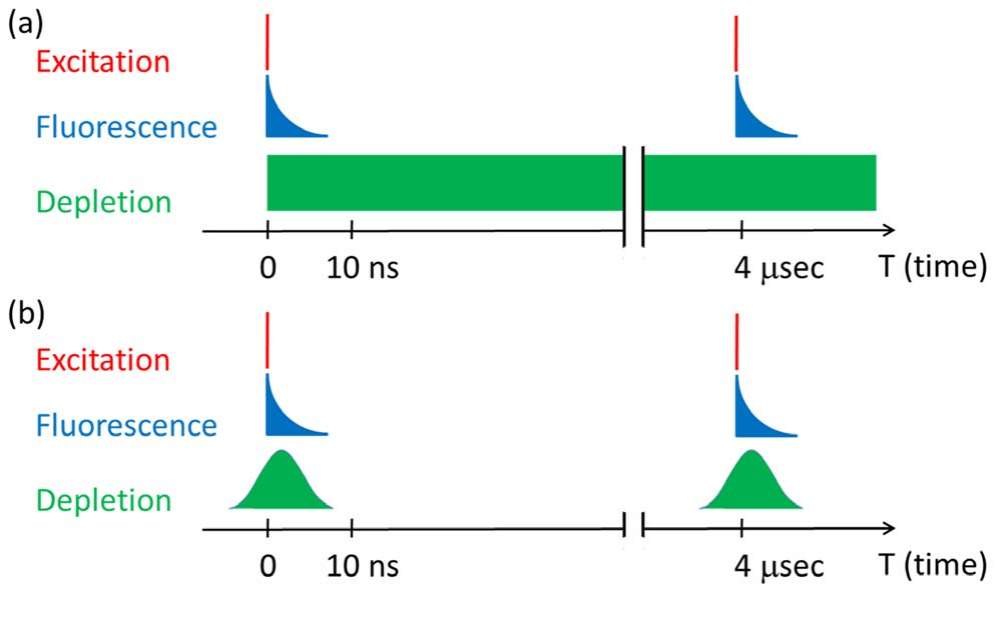
Temporal profile of the excitation pulse, fluorescence, and depletion beam. (a) In a CW STED configuration, the CW depletion beam at 532 nm illuminates the sample at all time. (b) In the modulated-CW STED configuration, the CW 532 nm laser is modulated by an acousto-optic Bragg cell to generate 10 ns pulses, which significantly reduce the laser exposure to the sample.

Although the lower repetition rate (0.25 MHz) increases the fluorescence yield, it creates an undesirable situation for CW-STED microscopy. As shown in Fig. 3a, while the excitation pulse is 4 µs apart, the fluorescence lifetimes of typical dyes are in the range of 3–5 ns. Therefore the CW depletion is only effective in the first 10 ns out of 4 µs. The situation causes unnecessary laser heating and damages by the 532-nm depletion beam. In this study, this issue was solved by passing the CW 532 nm beam through the acousto-optic Bragg cell, which was used to reduce the repetition rate of the Ti:sapphire laser. The Bragg cell pulse picker generates a 10-ns gate to pick up a single pulse from the pulse train of the Ti:sapphire laser. This 10-ns gate is very effective in producing a 10-ns pulse out of a CW laser beam. An additional advantage of this approach is that the 10-ns 532 nm pulse is automatically synchronized with the excitation beam because they pass through the same Bragg cell. Therefore, no additional synchronization equipment is needed. As indicated in Fig. 3b, the Bragg cell turns on the 532 nm depletion beam when the excitation pulse presents and turns off the 532 nm beam when the fluorescence has been depleted. With a repetition rate of 0.25 MHz, this approach reduced the power of the 532-nm beam on the sample by nearly 3 orders of magnitude. In the current study, only 0.11 mW of 532 nm beam was illuminated on the sample, in comparison to hundreds of mW typically used for CW-STED. [13] This modulation technique is particularly beneficial for CW STED when the pulsed excitation is used.

**Figure 4:**
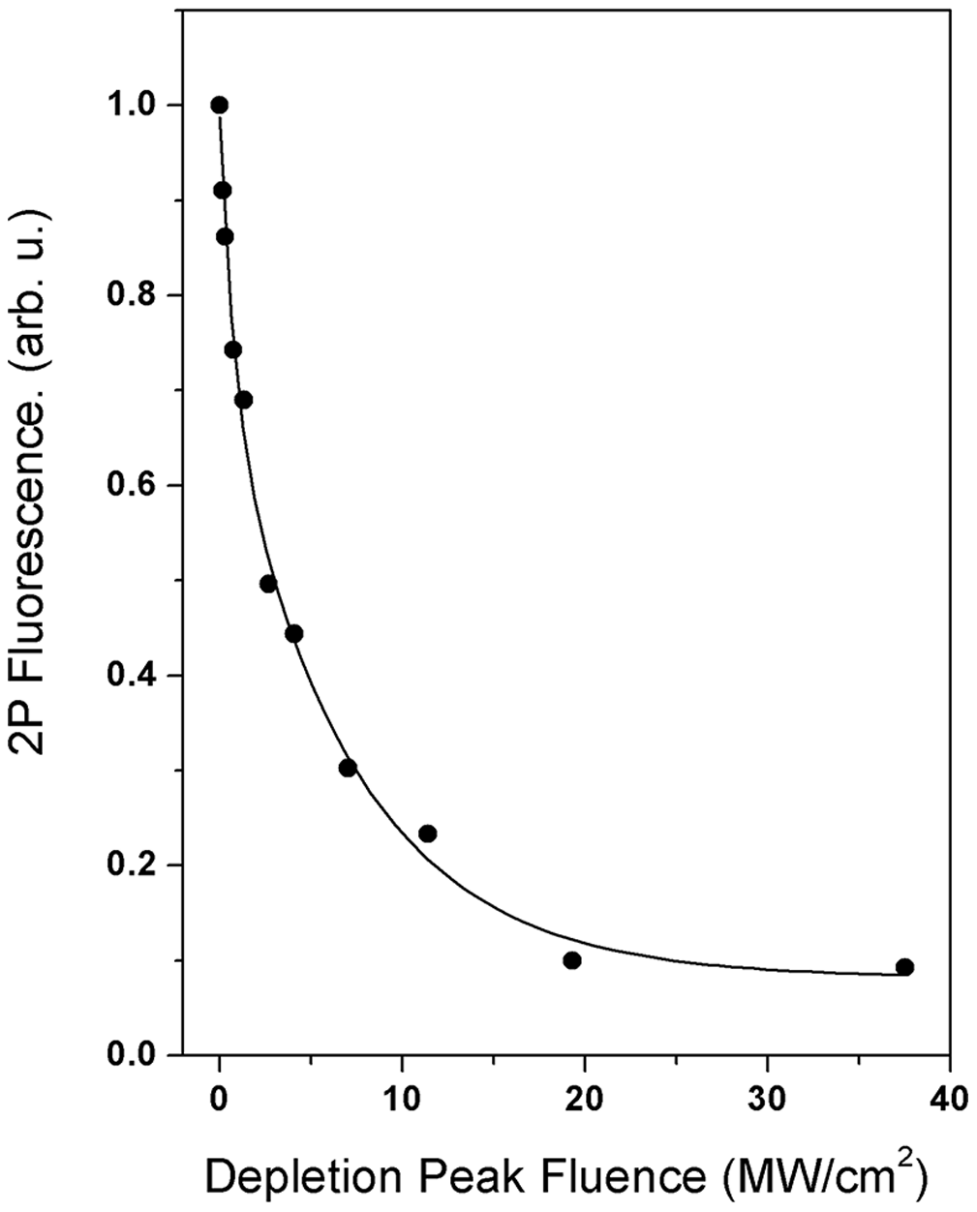
Two-photon excited fluorescence depletion of ATTO 425 depends on the 532 nm depletion beam.

The selection of fluorophores for the 2PE STED microscopy needs to meet the following criteria: (1) high 2P absorption cross sections in the 700–1000 nm region, (2) effective depletion of the fluorescence by the 532 nm laser beam, and (3) negligible excitation by the 532 nm laser beam. Because of the large selections of fluorophores, it is relatively easy to find one with a big 2P absorption cross section in the 700–1000 nm region. Criteria (2) and (3) are generally acting against each other. Although the wavelength of the depletion beam is red-shifted from a fluorophore’s single-photon absorption peak, the depletion beam is often capable of exciting the fluorophore due to its high input power. Tuning the depletion wavelength toward red, the excitation will decrease, but the efficiency of the depletion will also decrease. In the current study, the 532 nm beam used to pump the Ti:sapphire laser is fixed, so the fluorophore has to be selected to meet the aforementioned three criteria. Several fluorophores, including GFP, CFP, Alexa 488, and ATTO 425, have been tested, and ATTO 425 showed a promising result with good depletion efficiency and negligible excitation by the 532 nm beam. Fig. 4 shows the fluorescence intensity of ATTO 425 as vs. the fluence of 532 nm beam. Generally, fluorescence depletion better than 90% is needed to obtain a good STED image.

**Figure 5:**
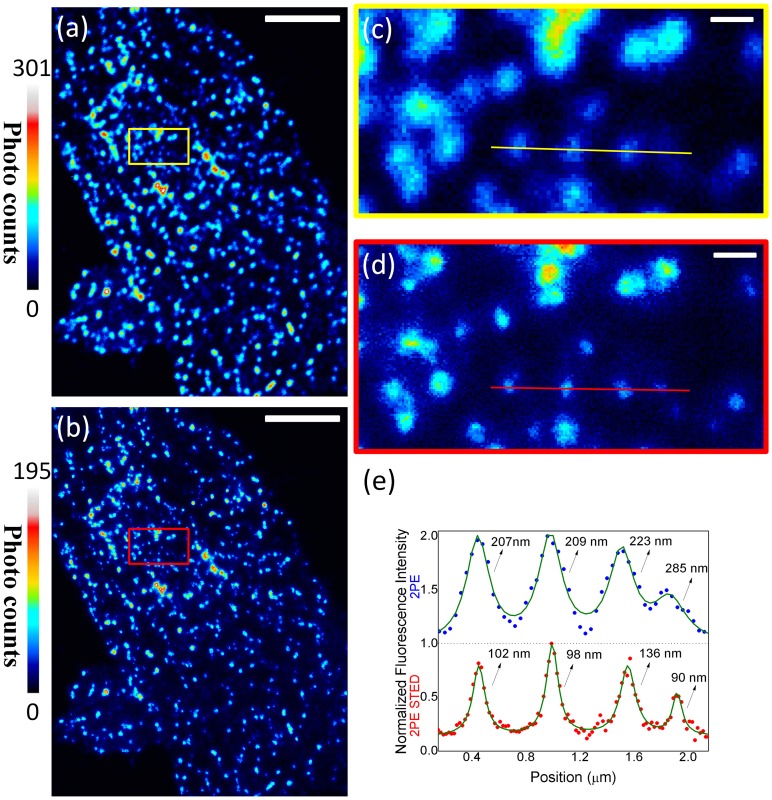
Images of ATTO 425-tagged clathrin in a CHO cell. (a) regular 2PE microscope and (b) the 2PE-STED microscope. (c) Magnified view of the marked area for the 2PE image. (d) Magnified view of the marked area for the 2PE-STED image. (e) Intensity profiles (scattering dots) of the 2PE and 2PE-STED images, and fitting with a Lorentzian function (solid lines) in (c) and (d). The scale bars are 5 µm in (a) and (b) and 400 nm in (c) and (d). The pixel sizes are 40 nm for (c) and 20 nm for (d).

Figs. 5a and 5b are images of ATTO 425-tagged clathrin in Chinese hamster ovary (CHO) cells imaged by a regular 2PE microscope and by the 2PE STED microscope, respectively. Figs. 5c and 5d are the magnified views of the marked areas in Figs. 5a and 5b, respectively. Fluorescence spots represent the clathrin-coated vesicles which have a size of approximately 50–200 nm under the electron microscope. [23] Fig. 5e shows the profiles of the cross sections indicated by the scattering dots in Figs. 5c and 5d. To compare the line profiles from 2PE and 2PE-STED images, we fitted the profiles with the Lorentzian function: **I** = **A**+**B**×**Γ**/(4(***x***–***x_c_***)^2^+**Γ** ^2^), where **A** and **B** are constants, ***x_c_*** is the center, and **Γ** is the width of the peak. [15] The regular 2PE microscope has a diffraction-limited excitation spot with width **Γ** of ∼210 nm. Consequently, all features observed in the regular 2PEF images (Figs. 2c and 2e) have width larger than 210 nm. With the 2PE STED microscope, however, clathrin-coated vesicles with a diameter less than 100 nm can be observed, which is more consistent with the sizes observed by electron microscopes.

The modulated-CW 2PE STED microscope has the benefits of both CW and pulse approaches: lower input power, simple optical scheme and no complicated synchronization. Additionally, it also takes advantages of fluorescence yield increasing. [22] We believe that this imaging technique can be improved toward an invasive superresolving tool deep in thick samples. Firstly, NIR CW laser beam has to be chosen taking into account long penetration depth. In this work, we used 532 nm laser beam as the depletion beam because it is readily available as the pumping source of a Ti:sapphire laser, though 532 nm laser beam brought much more antofluorescence and photobleaching. Secondly, laser scanning is a good strategy to acquire quick images of living cell or tissue, [15], [16] otherwise the acquisition time will be limited by piezo-scanning stage. Finally, the resolution of this modulated-CW 2PE STED can be also improved using time gating. [20].

## Conclusion

We presented a cost effective approach to carry out 2PE STED microscopy using a Ti:sapphire laser as the excitation beam and the CW 532 nm laser as the depletion beam. With the addition of an acoustic-optic Bragg cell to modulate the CW laser, we showed that the modulated-CW STED can take advantages of fluorescence yield increasing while simplify optical scheme. The sub-diffraction-limit resolution was demonstrated using ATTO 425-tagged clathrin-coated vesicles.

## References

[1] Lichtman JW, Conchello JA (2005) Fluorescence microscopy. Nat Methods 2: 910–919. 16299476 10.1038/nmeth817

[2] Erni R, Rossell MD, Kisielowski C, Dahmen U (2009) Atomic-Resolution Imaging with a Sub-50-pm Electron Probe. Phys Rev Lett 102: 4. 10.1103/PhysRevLett.102.096101 19392535

[3] Al-Amoudi A, Chang JJ, Leforestier A, McDowall A, Salamin LM, et al (2004) Cryo-electron microscopy of vitreous sections. Embo Journal 23: 3583–3588. 15318169 10.1038/sj.emboj.7600366 PMC517607

[4] Willig KI, Rizzoli SO, Westphal V, Jahn R, Hell SW (2006) STED microscopy reveals that synaptotagmin remains clustered after synaptic vesicle exocytosis. Nature 440: 935–939. 16612384 10.1038/nature04592

[5] Betzig E, Patterson GH, Sougrat R, Lindwasser OW, Olenych S, et al (2006) Imaging intracellular fluorescent proteins at nanometer resolution. Science 313: 1642–1645. 16902090 10.1126/science.1127344

[6] Hess ST, Girirajan TPK, Mason MD (2006) Ultra-high resolution imaging by fluorescence photoactivation localization microscopy. Biophys J 91: 4258–4272. 16980368 10.1529/biophysj.106.091116 PMC1635685

[7] Rust MJ, Bates M, Zhuang XW (2006) Sub-diffraction-limit imaging by stochastic optical reconstruction microscopy (STORM). Nat Methods 3: 793–795. 16896339 10.1038/nmeth929 PMC2700296

[8] Gustafsson MGL (2005) Nonlinear structured-illumination microscopy: Wide-field fluorescence imaging with theoretically unlimited resolution. Proc Natl Acad Sci U S A 102: 13081–13086. 16141335 10.1073/pnas.0406877102 PMC1201569

[9] Leung BO, Chou KC (2011) Review of Super-Resolution Fluorescence Microscopy for Biology. Appl Spectrosc 65: 967–980. 21929850 10.1366/11-06398

[10] Hell SW, Wichmann J (1994) Breaking the Diffraction Resolution Limit by Stimulated-Emission - Stimulated-Emission-Depletion Fluorescence Microscopy. Opt Lett 19: 780–782. 19844443 10.1364/ol.19.000780

[11] Klar TA, Jakobs S, Dyba M, Egner A, Hell SW (2000) Fluorescence microscopy with diffraction resolution barrier broken by stimulated emission. P Natl Acad Sci USA 97: 8206–8210. 10.1073/pnas.97.15.8206 PMC26924 10899992

[12] Donnert G, Keller J, Medda R, Andrei MA, Rizzoli SO, et al (2006) Macromolecular-scale resolution in biological fluorescence microscopy. Proc Natl Acad Sci U S A 103: 11440–11445. 16864773 10.1073/pnas.0604965103 PMC1518808

[13] Willig KI, Harke B, Medda R, Hell SW (2007) STED microscopy with continuous wave beams. Nature Methods 4: 915–918. 17952088 10.1038/nmeth1108

[14] Li QF, Wu SSH, Chou KC (2009) Subdiffraction-Limit Two-Photon Fluorescence Microscopy for GFP-Tagged Cell Imaging. Biophysical Journal 97: 3224–3228. 20006960 10.1016/j.bpj.2009.09.038 PMC2793354

[15] Bethge P, Chereau R, Avignone E, Marsicano G, Nagerl UV (2013) Two-Photon Excitation STED Microscopy in Two Colors in Acute Brain Slices. Biophysical Journal 104: 778–785. 23442956 10.1016/j.bpj.2012.12.054 PMC3576543

[16] Takasaki KT, Ding JB, Sabatini BL (2013) Live-Cell Superresolution Imaging by Pulsed STED Two-Photon Excitation Microscopy. Biophysical Journal 104: 770–777. 23442955 10.1016/j.bpj.2012.12.053 PMC3576532

[17] Bianchini P, Harke B, Galiani S, Vicidomini G, Diaspro A (2012) Single-wavelength two-photon excitation-stimulated emission depletion (SW2PE-STED) superresolution imaging. Proceedings of the National Academy of Sciences of the United States of America 109: 6390–6393. 22493221 10.1073/pnas.1119129109 PMC3340040

[18] Ding JB, Takasaki KT, Sabatini BL (2009) Supraresolution Imaging in Brain Slices using Stimulated-Emission Depletion Two-Photon Laser Scanning Microscopy. Neuron 63: 429–437. 19709626 10.1016/j.neuron.2009.07.011 PMC2756148

[19] Moneron G, Hell SW (2009) Two-photon excitation STED microscopy. Opt Express 17: 14567–14573. 19687936 10.1364/oe.17.014567

[20] Vicidomini G, Moneron G, Han KY, Westphal V, Ta H, et al (2011) Sharper low-power STED nanoscopy by time gating. Nature Methods 8: 571–U575. 21642963 10.1038/nmeth.1624

[21] Torok P, Munro PRT (2004) The use of Gauss-Laguerre vector beams in STED microscopy. Opt Express 12: 3605–3617. 19483892 10.1364/opex.12.003605

[22] Donnert G, Eggeling C, Hell SW (2007) Major signal increase in fluorescence microscopy through dark-state relaxation. Nature Methods 4: 81–86. 17179937 10.1038/nmeth986

[23] McMahon HT, Boucrot E (2011) Molecular mechanism and physiological functions of clathrin-mediated endocytosis. Nature Reviews Molecular Cell Biology 12: 517–533. 21779028 10.1038/nrm3151

